# Cell-based reference samples designed with specific differences in microRNA biomarkers

**DOI:** 10.1186/s12896-018-0423-4

**Published:** 2018-03-20

**Authors:** P. Scott Pine, Steven P. Lund, Sanford A. Stass, Debra Kukuruga, Feng Jiang, Lynn Sorbara, Sudhir Srivastava, Marc Salit

**Affiliations:** 1 Joint Initiative for Metrology in Biology, National Institute of Standards and Technology, 443 Via Ortega, Stanford, CA 94305 USA; 2 Statistical Engineering Division, National Institute of Standards and Technology, Gaithersburg, MD 20899 USA; 3 Department of Pathology, University of Maryland School of Medicine, Baltimore, MD 21201 USA; 4 Division of Cancer Prevention, National Cancer Institute, Rockville, MD 20850 USA

**Keywords:** Reverse transcription PCR (RT-PCR), microRNA (miRNA), Reference samples, Biomarkers, Performance assessment, Measurement assurance

## Abstract

**Background:**

We demonstrate the feasibility of creating a pair of reference samples to be used as surrogates for clinical samples measured in either a research or clinical laboratory setting. The reference sample paradigm presented and evaluated here is designed to assess the capability of a measurement process to detect true differences between two biological samples. Cell-based reference samples can be created with a biomarker signature pattern designed in silico. Clinical laboratories working in regulated applications are required to participate in proficiency testing programs; research laboratories doing discovery typically do not. These reference samples can be used in proficiency tests or as process controls that allow a laboratory to evaluate and optimize its measurement systems, monitor performance over time (process drift), assess changes in protocols, reagents, and/or personnel, maintain standard operating procedures, and most importantly, provide evidence for quality results.

**Results:**

The biomarkers of interest in this study are microRNAs (miRNAs), small non-coding RNAs involved in the regulation of gene expression. Multiple lung cancer associated cell lines were determined by reverse transcription (RT)-PCR to have sufficiently different miRNA profiles to serve as components in mixture designs as reference samples. In silico models based on the component profiles were used to predict miRNA abundance ratios between two different cell line mixtures, providing target values for profiles obtained from in vitro mixtures. Two reference sample types were tested: total RNA mixed after extraction from cell lines, and intact cells mixed prior to RNA extraction. MicroRNA profiling of a pair of samples composed of extracted RNA derived from these cell lines successfully replicated the target values. Mixtures of intact cells from these lines also approximated the target values, demonstrating potential utility as mimics for clinical specimens. Both designs demonstrated their utility as reference samples for inter- or intra-laboratory testing.

**Conclusions:**

Cell-based reference samples can be created for performance assessment of a measurement process from biomolecule extraction through quantitation. Although this study focused on miRNA profiling with RT-PCR using cell lines associated with lung cancer, the paradigm demonstrated here should be extendable to genome-scale platforms and other biomolecular endpoints.

**Electronic supplementary material:**

The online version of this article (10.1186/s12896-018-0423-4) contains supplementary material, which is available to authorized users.

## Background

The translation of a biomarker from discovery to clinical practice involves a transition from the research-oriented approach of the discovery laboratory to a procedure in an accredited clinical laboratory. However, most published cancer biomarkers never make it into clinical practice [1], with recent work highlighting the irreproducibility of results [2]. Demonstrating that methodology is accurately transferred is critical to the biomarker validation process. This process can be conceptualized by multiple phases of biomarker development [3]. Within the framework of the Early Detection Research Network (EDRN) of the National Cancer Institute [4], we are developing a measurement assurance paradigm for the first phase of this process, *preclinical exploratory studies*. Quality measurements during this phase are essential for the success of all subsequent phases of biomarker development.

Preclinical studies to develop cancer biomarkers typically involve comparing measurement results from tumor tissue to normal tissue and identifying features that distinguish the two classes. Reference samples, with known differences, enable laboratories to demonstrate their ability to detect potential biomarkers. Ideally, these biomarkers would be measured in one or more biofluids and provide a means for non-invasive screening at the earliest stages for the detection of cancer. Two EDRN laboratories are assessing microRNAs (miRNAs) found in sputum as potential biomarkers, which is the context for designing mixture reference samples described in the present paper.

MicroRNAs are a class of small non-protein-coding RNAs that regulate the expression of hundreds of target genes, controlling biological functions involved in differentiation and development [5]. Dysregulation of miRNA expression may contribute to cancer development and progression [6]. Therefore, miRNAs are potential candidates as useful biomarkers.

Three cell lines with relatively different expression profiles for five miRNAs of interest were assessed as potential mixture components to create a pair of reference samples to act as surrogates for sputum samples measured in a non-invasive screening assay for lung cancer. Lung cancer cell lines from the NCI-H series (H226, H358, and H460) [7] were profiled for the expression of miR-21 [8], miR-126 [9], miR-210 [10], miR-375 [11], and miR-486 [12]. These miRNA were selected based upon their putative roles in cancer and previous screening results of the BDL that found that three of these miRNA (miR-21, miR-210, and miR-375) were *overexpressed* and two (miR-126 and miR-486) were *underexpressed* when comparing lung cancer and normal tissue, providing a potential panel of biomarkers for early detection [13, 14].

The miRNA profile of each reference sample need not mimic a particular biological state (i.e., normal or disease), but should provide the ability to assess technical performance of the measurement process used to discriminate based on the particular set of miRNAs of interest. Ideally, these reference samples should possess the following properties: 1) express all or most of the biomarkers of interest; 2) be regenerable for use over time as a process control; 3) be amenable to identical processing as clinical samples; 4) contain well-established relative differences in the biomarkers of interest; and 5) be commutable between measurement systems. Such samples could be used to assess repeatability within, and reproducibility between, laboratories for the entire measurement process.

**Fig. 1:**
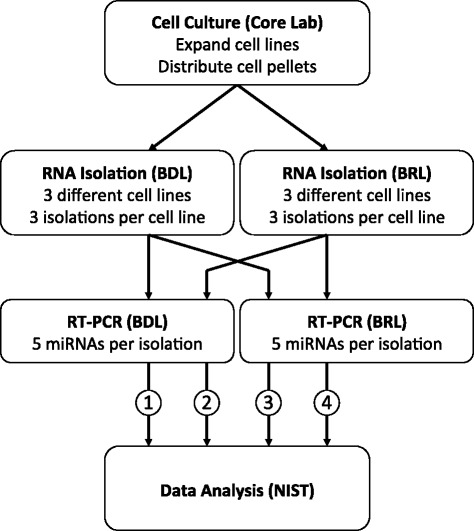
Experimental workflow with sample crossover for miRNA profiling of cell lines providing four different measurement scenarios, indicated by pathways 1–4, for comparison of laboratory effects (see also Additional file 1: Table S2)

In the first part of this study, a “crossover” design was used to assess reproducibility of miRNA profiles and parse out factors contributing to variability (see Fig. 1). Two different EDRN sites participated: one a biomarker development laboratory (BDL) engaged in clinical research; and the other a Clinical Laboratory Improvement Amendments (CLIA) compliant and College of American Pathology (CAP) accredited biomarker reference laboratory (BRL). Each laboratory used the same source of cells, which were grown in the core cell facility of the BRL. A standard operating procedure for RNA isolation was developed by the BRL for both laboratories, meeting the CLIA standards of the BRL. Each site measured the same set of miRNAs of interest using reverse transcription PCR (RT-PCR). Both sites measured these miRNAs in the total RNA samples they isolated, as well as those isolated at the other site, on three separate occasions. Datasets from each site were subsequently analyzed at the National Institute for Standards and Technology (NIST). This design permits evaluation of interlaboratory variability due to either RNA extraction or miRNA measurement.

**Fig. 2:**
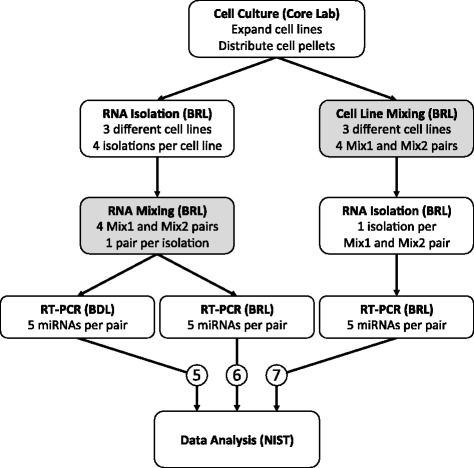
Experimental workflow for evaluating use of mixtures introduced at different stages (shaded boxes) of the miRNA profiling process with three different measurement scenarios, indicated by pathways 5–7, providing source material comparisons (see also Additional file 1: Table S2)

To inform the design for the two reference samples, the miRNA measurements for each cell line were used to predict the ratio of miRNA abundance between samples formed by mixing the component cell lines in different proportions [15, 16]. To determine whether the mixture design was fit-for-purpose, the predicted ratios were compared with corresponding measurements from reference mixture samples prepared by two different methods: mixing total RNA extracted from the cell lines in the BRL, and mixing the cell lines before total RNA isolation by the BRL (see Fig. 2). The first approach provides RNA samples suitable for assessing each lab’s ability to measure differential expression using their RT-PCR process. The second design provides reference samples suitable for assessing the entire measurement process of a lab.

## Methods

### Cell culture

Cell Lines were obtained from the American Type Culture Collection (ATCC®), Manassas, VA. H226 (ATCC# CRL-5826), H358 (ATCC# CRL-5807), and H460 (ATCC# HTB-177) were the cell lines selected by the biomarker discovery laboratory (BDL) [13, 14]. The core cell culture facility for the biomarker reference laboratory (BRL) expanded the cells and provided frozen cell pellets in RNAlater (Life Technologies, Grand Island, NY). Briefly, cells were thawed fresh at the lowest possible passage (< 25 passages) for each new batch, using the standard thawing method in 37 °C and dilution with culture medium into a new culture flask. The day after thawing, medium was replaced to completely remove residual DMSO. Cells did not exceed 80 to 85% confluence during cell expansion. T-225 flasks were seeded with 2.5 to 5.0 million cells, dependent on each cell line’s growth characteristics. Cells were harvested using the standard PBS wash and trypsinization procedure. Recovered cells were counted 3X with an automated cell counter. For the initial profiling phase, a sufficient number of cells were expanded to provide for three separate isolations per lab. Separate expansions were performed to provide cells for the RNA mixture experiments and the cell line mixture experiments.

### RNA isolation

The BRL developed the following standard operating procedures for the isolation of total RNA enriched for miRNA from the cell pellets using the Qiagen miRNeasy Mini Kit. Briefly, cells are lysed in a denaturing buffer containing phenol / guanidine-thiocyanate designed to inhibit RNases to ensure the purification of intact RNA from cellular DNA and proteins. Samples are homogenized using the QIAShredder columns. Ethanol is then added to the sample to precipitate the RNA, and the sample is applied to an RNeasy mini spin column, where the RNA binds to the silica-based membrane. After washing contaminants away, RNA is eluted from the membrane in RNase-free water. Each lab performed three separate RNA isolations on different days on each component cell line.

### RNA mixtures

For total RNA mixtures, a 3-component design with a 1:1 component and two reciprocal 3:1 components were used. Total RNA isolated by the BRL was quantified using an Agilent BioAnalyzer and mixed by mass in the following proportions: Mix1 (1:3:1) and Mix2 (3:1:1), using H226, H358, and H460, respectively. In silico modeling was used to design the mixtures, selecting the cell line mixing proportion to use for each component that provided a useful distribution of ratios across the dynamic range (see **Results**).

### Cell line mixtures

For cell line mixtures, intact cells were collected during log phase growth and counted in triplicate with an automated cell counter. Cells were then mixed (count:count:count) in the same proportions as the RNA mixtures: Mix1 (1:3:1) and Mix2 (3:1:1), using H226, H358, and H460, respectively.

### RT-PCR measurement

For miRNA profiling of cell lines, each lab measured the RNA isolated within their lab as well as the RNA isolated by the other lab using RT-PCR. Human (Hsa-) specific kits (Applied Biosystems, Foster City, CA) for miR-16 (#000391), miR-21 (#000397), miR-126 (#002228), miR-210 (#000512), miR-486 (#001278), miR-375 (#000564) were used for reversed transcription to cDNA. The TaqMan miRNA assays were performed using an ABI 7900 HT (Applied Biosystems, Foster City, CA) in the BRL and an IQ5 Multicolor Real-Time PCR Detection System (Biorad Laboratories, Hercules, CA) in the BDL. The same RT-PCR protocol was used for miRNA profiling of mixtures from RNA components or cell line components.

### In silico modeling

Each analyte (among miR-21, miR-126, miR-210, miR-375, and miR-486) was modeled independently according to the following analysis. From the individual cell lines, let *Y*_*ceip*_ denote the average of three Cq measurements from within a single plate for RNA extracted from cell line *c* (*c* = 1, 2, 3; 1 = H226, 2 = H358, 3 = H460) during extraction replicate *e* (*e* = 1, 2, 3) at isolation lab *i* (*i* = 1, 2; 1 = BDL, 2 = BRL) and measured at PCR lab *p* (*p* = 1, 2; 1 = BDL, 2 = BRL). Under the assumption that miRNA abundance should be additive and linear, we can predict a Cq value for a hypothetical sample reflecting a designed mixture of the component cell line samples using the following equation:

1 $$ {\widehat{M}}_{meip}={-\mathit{\log}}_2{\sum}_{c=1}^3{2}^{-{Y}_{ceip}}\ast {\phi}_{cm} $$

where ${\widehat{M}}_{meip}$ is the predicted Cq measurement at PCR lab *p* of designed mixture *m* (*m* = 1, 2) hypothetically created by combining the samples from isolation replicate *e* at isolation lab *i* at relative concentrations of *ϕ*_1*m*_, *ϕ*_2*m*_, and *ϕ*_3*m*_ for component cell lines H226, H358 and H460, respectively. We refer to each of the four combinations of isolation lab and PCR lab as a different measurement process.

### Statistical modeling

Data collected throughout the described experiments were analyzed in a Bayesian framework to evaluate the uncertainty related to average Cq values or their differences (e.g., average ΔCq values). For each target miRNA in turn, the observed Cq values were analyzed using mixed effects models that include a fixed mean for each of 18 distinct combinations (see Additional file 1: Table S1) of source material, measurement method and target, as well as random effects for plate and isolation. The modeled average Cq values were used to evaluate and compare average ΔCq values (Mix1–Mix2) for seven different measurement scenarios (see Additional file 1: Table S2).

Modeling results are presented in terms of marginal likelihoods, rather than posterior probabilities, to reduce the influence of subjective choices for prior distributions. A marginal likelihood reflects the chance of the observed data occurring assuming a particular value for a model parameter of interest (e.g., average Cq, average ΔCq, or difference in average ΔCq), after integrating across the modeled distribution of all other model parameters. We examine peak shapes formed by plotting marginal likelihood as a function of the value of the parameter of interest to assess whether or not particular factors appear to contribute to bias in these results. This approach is used to assess differences in Cq responses between samples (Mix1 vs. Mix2), laboratories (BDL vs. BRL), or source material (in silico vs. RNA vs. cells). In these plots, a value of 0 along the x-axis indicates “no difference” for the comparison while deviation from zero indicates a possible bias.

To examine the sensitivity of the reported results to modeling choices, the entire analysis was repeated using three model perturbations. All models were fit using Markov Chain Monte Carlo (MCMC) via the R package *rjags* [17, 18]. MCMC is a computational tool commonly used in Bayesian analysis that relies on simulation over many iterations to approximate closed-form computations that are difficult to evaluate because they involve complicated probability distributions. An introduction to Bayesian analysis in the context of the presented experiment and additional details of the three considered models are provided in the Additional file 1. The interested reader may also find a vast collection of more general references for Bayesian theory and practice, including the use of MCMC; see for instance [19]. Datasets and R code used to conduct the analysis are provided in Additional files 2, 3, 4 and 5.

## Results

### Relative miRNA expression levels in different cell lines

The expression profiles of five miRNAs of interest (miR-21, miR-126, miR-210, miR-375, and miR-486) [13, 14] and one control (miR-16) across three different cell lines were measured by both the BRL and BDL. Each laboratory performed three separate RNA isolations per cell line, split their samples and shared them with the other site. Each laboratory then used their own RT-PCR methods to measure the miRNA profiles of both sets of samples. This cross-over approach was used to determine if robust differences in miRNA abundance were consistently observed among the four possible measurement processes resulting from all combinations of isolation laboratory and PCR laboratory (see Fig. 1). Throughout this paper, the term Cq value refers to the average Cq (taken over 3 replicate wells) for a given miRNA in a given cell line, evaluated for each isolation and each process.

**Fig. 3:**
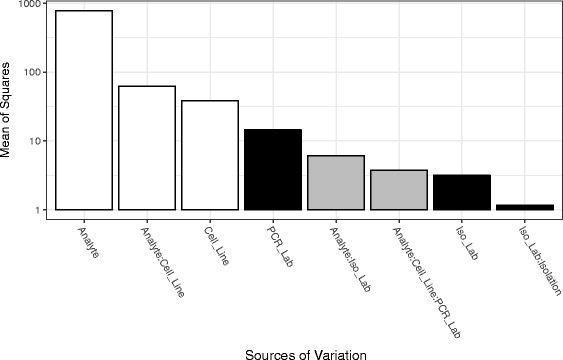
Sources of variance in the miRNA profiling dataset. An analysis of variance in R [17] was used to identify the major sources of variability in the miRNA profiling of three cell lines by two laboratories using a cross-over design (see Fig. 1). A five-way ANOVA, testing all possible interactions, was applied to determine the contribution to variance by analyte, cell line, isolation laboratory, isolation process, and PCR laboratory. The input data for the model was the Cq for each of the four measurement processes for three isolations. The mean of squares is plotted on the y-axis on a log10 scale and is a measure of the average contribution of each factor or interaction to the variability in the experiment. Factors or interactions with a mean of squares less the one are not included. White bars correspond to biological factors or interactions, black bars correspond to laboratory factors or interactions, and grey bars correspond to interactions between biological and laboratory factors

An analysis of variance (ANOVA) was performed on the Cq values in phase 1 (RNA extracted from pure cell lines). Fig. 3 shows the factors with the greatest mean squares (i.e., sum of squares divided by corresponding degrees of freedom). As expected, the biologically relevant factors of analyte, cell line and their interaction are the three most prominent components of variability. The confounding factor with the greatest attributed variability in Cq values was which lab conducted PCR analysis.

When evaluating log abundance ratios (i.e., differences in Cq values) across cell lines (or cell line mixtures), only the interactions involving cell line will matter. This is because effects that remain constant across cell lines will cancel when taking the difference (e.g., increasing the Cq value by 0.5 for all PCR measurements conducted at BRL will not impact the estimated log abundance ratio of a given miR between any two cell lines). From this perspective, the most substantial source of confounding variability is the factor associated with the interaction among analyte, cell line and PCR lab, which contributes, on average, about an order of magnitude less variability than do any of the biologically-relevant factors.

**Fig. 4:**
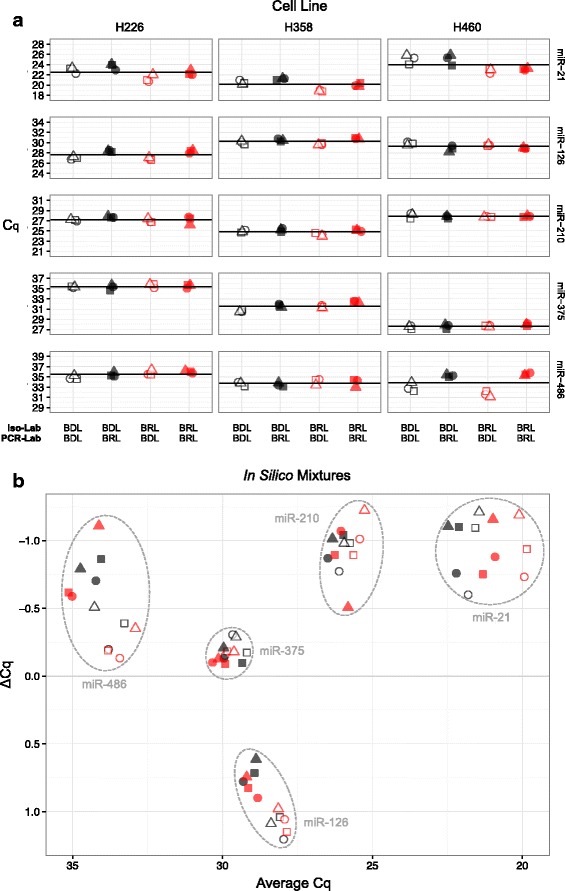
Expression profiling of miRNAs of interest in candidate cell lines. Panel **a** shows the Cq values for each miRNA and measurement scenario (see Fig. 1). Total RNA samples were isolated in the BDL (black symbols) and BRL (red symbols) on three separate occasions (circles, squares, and triangles). RT-PCR results are from BDL (open symbols) and BRL (filled symbols), where the black line corresponds to the mean Cq for all isolation and PCR combinations. Horizontal jitter added for display purposes. Panel **b** is a Bland-Altman plot (or difference plot) of relative abundance, where ΔCq = Mix1 – Mix2 and the Cq values are predicted in silico from PCR measurements made in either the BDL (open symbols) or BRL (filled symbols) using pure total RNA profiles of component cells from each isolation (circles, squares, and triangles) performed in the BDL (black symbols) or BRL (red symbols) using Eq. 1 and the mixture design described in the Results. X-axis is in reverse order to indicate direction of relative abundance (i.e., lower Cq values indicates more starting material). Similarly, y-axis is in reverse order to indicate that a negative ΔCq corresponds to more starting material in Mix1 than Mix2

Figure 4a shows there was concordance among the four processes with respect to which of the miRNAs of interest were more abundant (lower Cq) in each cell line: H358 cells had the highest expression for miR-21 and miR-210; H226 cells had the highest expression of miR-126; and H460 cells had the highest expression of miR-375. Both H358 and H460 expressed similar levels of miR-486. MiR-21 also appeared more abundant in samples isolated by the BRL than from the BDL (red symbols and black symbols, respectively). Both miR-126 and miR-486 showed more difference with respect to which lab performed the PCR measurements, BDL (open symbols) or BRL (filled symbols). Both miR-375 and miR-486 had Cq values around 35 in H226 cells, a value indicating either single molecule detection or noise [20], indicating minimal, if any signal contribution to the final mixtures. Overall, expression levels were sufficiently different between H226, H358, and H460 to proceed to in silico modeling for mixtures. ANOVA results of laboratory factors after the datasets are parsed by analyte and cell line are available in Additional file 6.

Two different mixture designs were selected on the basis of iterative in silico modeling via Eq. 1. The results from each iteration were viewed as a Bland-Altman plot or difference plot [21], where the predicted measurement difference between two mixtures is plotted as a function of the predicted average of measurements for both mixture. Mixture fractions of *ϕ*_11_ = 0.2, *ϕ*_21_ = 0.6, *ϕ*_31_ = 0.2 were selected for mixture design 1 (Mix1); and *ϕ*_12_ = 0.6, *ϕ*_22_ = 0.2, *ϕ*_32_ = 0.2 for mixture design 2 (Mix2). This design predicted a useful distribution of analyte abundances across the dynamic range as well as ΔCq (Mix1 – Mix2) between the samples ranging from − 1 to 1, i.e., two-fold up and down as shown in Fig. 4b.

Each measurement process demonstrated a consistent rank order of miRNA with respect to average Cq. Although the smallest difference in that dimension was observed between miR-126 and miR-375, the mixing design still predicted a useful separation between those two miRNAs with respect to ΔCq. The isolation lab differences observed with miR-21 in Fig. 4a produce a similar trend in Fig. 4b for the modeled mixtures, where the samples isolated in the BRL are predicted to be more abundant (lower average Cq) than those from the BDL. Because this trend was similar for each cell line, the modeled ΔCq are nearly the same (see also Fig. 9, In Silico). The trends for the PCR lab differences observed in Fig. 4a for miR-126 and miR-486 vary across combinations of cell line and analyte (see ANOVA results in Fig. 3 corresponding to interaction among analyte, cell line and PCR lab), so the modeling predicts the average of both Cq and ΔCq will be affected (see also Fig. 9, In Silico). The modeled mixtures, as shown in Fig. 4b, provided a sufficiently unique pattern of differences to proceed with preparation of actual mixtures of total RNA.

### Reference samples of total RNA mixtures

**Fig. 5:**
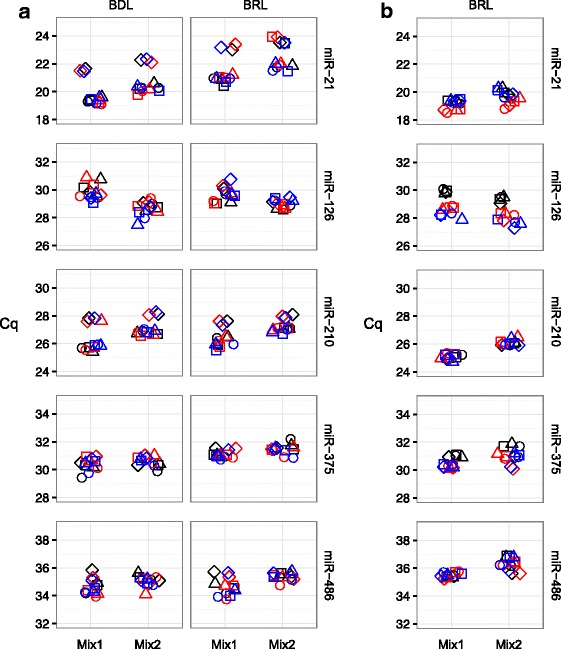
Expression profiling of miRNAs of interest in RNA mixtures. Panels **a** and **b** show the Cq values for total RNA mixtures and cell line mixtures, respectively. Mix1 and Mix2 were prepared volume:volume:volume, normalized for concentration, using pure total RNA isolated in the BRL (see Fig. 2, scenarios 5 and 6) or count:count:count from intact cells grown in the core facility (see Fig. 2, scenario 7) on four separate occasions (circles, squares, triangles, and diamonds). Each set of RNA mixture pairs (e.g. Mix1 circles and Mix2 circles) were split and measured in triplicate plates (black, red, and blue) by RT-PCR in both the BDL and BRL. For the cell line mixtures, only the BRL extracted RNA from each set of cell line mixture pairs and then measured by RT-PCR in triplicate plates. Horizontal jitter added for display purposes

Four separate total RNA isolations per cell line from separate expansions were performed in the BRL and each isolation was used to prepare a single Mix1 and Mix2 pair (see Fig. 2) using the cell line mixture fractions selected from the in silico modeling. Mixture samples were each measured in triplicate at both PCR labs (see Fig. 5a). Both PCR laboratories detected a lower amount of miR-486 in Mix1 and lower amounts of miR-21 and miR-210 in both RNA mixtures derived from the fourth isolation (diamond symbols). This observed effect could be attributable to differences in growth conditions for the cell expansion used for the fourth isolation. However, the observed differences between Mix1 and Mix2 remained comparable with the sample design. For example, the average (± SD) ΔCq for three replicate plates of miR-21 measured in the BDL was − 1.03 ± 0.11, − 0.67 ± 0.16, − 0.85 ± 0.12, and − 0.69 ± 0.15 for isolations 1, 2, 3 and 4, respectively.

**Fig. 6:**
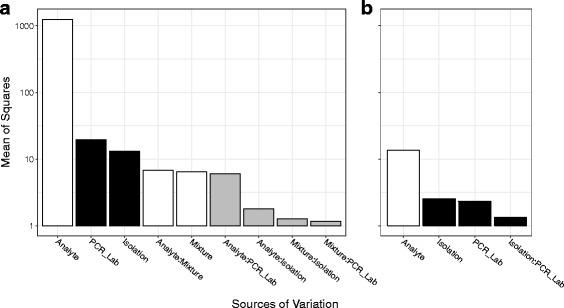
Sources of variance in the total RNA mixture dataset. An analysis of variance in R [17] was used to identify the major sources of variability in measuring 4 RNA mixture pairs by two laboratories (see Fig. 2, pathways 5 and 6). A four-way ANOVA, testing all possible interactions, was applied to determine the contribution to variance by analyte, mixture, isolation process, and PCR laboratory. In panel a, the input data for the model was the Cq for each of the miRNA measured by each PCR laboratory for each mixture prepared from four isolations (see Fig. 5, panel **a**). In panel **b**, the input data was the ΔCq between each mixture from the same dataset. The mean of squares is plotted on the y-axis on a log10 scale and is a measure of the average contribution of each factor or interaction to the variability in the experiment. Factors or interactions with a mean of squares less the one are not included. White bars correspond to biological factors or interactions, black bars correspond to laboratory factors or interactions, and grey bars correspond to interactions between biological and laboratory factors

An ANOVA was performed on the Cq values in phase 2 (RNA mixtures). In this phase, all isolations occurred at the BRL, which precludes further assessments of variability due to isolation lab. Figure 6a shows the factors with the greatest mean of squares. Analyte was the most prominent component of variability. The confounding factors corresponding to which lab conducted PCR analysis and differences among the four replicate isolations were the next most prominent sources of variability. The most substantial sources of confounding variability with respect to evaluating log abundance ratios across mixtures are the interactions between mixture and isolation and between mixture and PCR lab. The degree of variability attributed to mixture and the interaction between mixture and analyte are largely controlled by the designed abundance ratios resulting from the chosen cell line proportions for each mixture. Figure 6b corresponds to an ANOVA conducted on ΔCq (Mix1 – Mix2) values. The designed differences in ΔCq values across the five analytes represent the most prominent component of variability.

### Reference samples from cell line mixtures

To test the feasibility of using cell lines as surrogate samples, the three component cell lines were combined in the previously provided Mix1 and Mix2 proportions based upon cell counts to create four separate mixture pairs of cell suspensions. The mixture samples were then processed in triplicate by the BRL in the same manner as cells in a clinical specimen (see Fig. 5b). A PCR plate effect was observed for miR-21 and miR-126 when mixtures were considered separately. However, the observed differences between Mix1 and Mix2 remained comparable with the sample design. For example, the average (± SD) ΔCq for four isolations of miR-126 measured in the BRL was 0.56 ± 0.22, 0.55 ± 0.26, and 0.55 ± 0.30, for plates 1, 2, and 3, respectively.

### Detecting differences between reference sample pairs

**Fig. 7:**
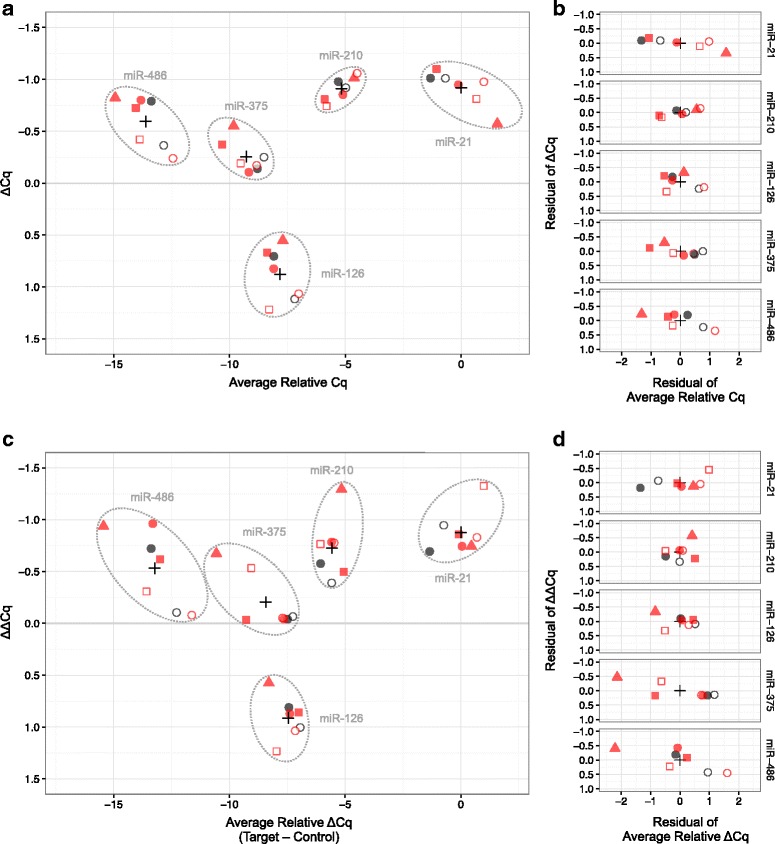
Average difference in relative abundance between Mix1 and Mix2 for all measurement scenarios (see Figs. 1 and 2). Panel **a** is the Bland-Altman plot of relative abundance where ΔCq = Mix1 – Mix2 and Cq values are rescaled relative to the average miR-21 value. Panel **c** is the Bland-Altman plot of relative abundance of Mix1 and Mix2 compared with ΔΔCq result obtained when using miR-16 as the control [22]. Panels **b** and **d** display residuals from panels **a** and **c**, respectively (i.e., data for each miRNA are re-centered around corresponding black cross seen in panels **a** and **c**, respectively). The black cross denotes center of all values for a particular method of calculation (either ΔCq or ΔΔCq) for each miRNA. Summarized values for each miRNA were compared for each analysis method across the three samples: in silico mixes, RNA mixes, and cell mixes (circles, squares, and triangles, respectively). RNA isolations were performed in either the BDL (black symbols) or BRL (red symbols) and analyzed by RT-PCR in the BDL (open symbols) or BRL (filled symbols). Y-axis is in reverse order to indicate that a negative ΔCq corresponds to more starting material in Mix1 than Mix2

Figure 7 shows the results for measuring the difference between samples applied to all data derived from either in silico modeled mixes, RNA mixes, or cell mixes using two different methods. The delta Cq (ΔCq) method was used to directly measure the relative difference between Mix1 and Mix2 using the mean Cq values from each miRNA target summarized by process (isolation lab, PCR lab, and sample type). In the delta-delta Cq (ΔΔCq) method [22], each Cq value for a miRNA target was adjusted to produce a ΔCq by subtracting the Cq value corresponding to miR-16 derived from the same sample (i.e., subject to the same experimental conditions of isolation, PCR lab) and measured on the same plate. The miR-16 adjusted values for each mixture were then compared (ΔΔCq). Figure 7a shows the distribution of relative abundance and differences in a plot similar to Fig. 4b, with the x-axis normalized to the average miR-21 Cq value. Examining the residuals highlights there is more variability in the average relative Cq than in the ΔCq (Fig. 7b). This is consistent with the ANOVA results shown in Figs. 3 and 6, which suggest the most prominent sources of confounding variation will cancel out when taking differences to compute ΔCq values. Additionally, for these data, using miR-16 as a control (ΔΔCq method) did not substantially reduce variability among multiple measurements of log abundance ratios compared to those derived directly from the Cq values of miRNA targets (Fig. 7, panels c and d compared to panels a and b, respectively).

## Discussion

**Fig. 8:**
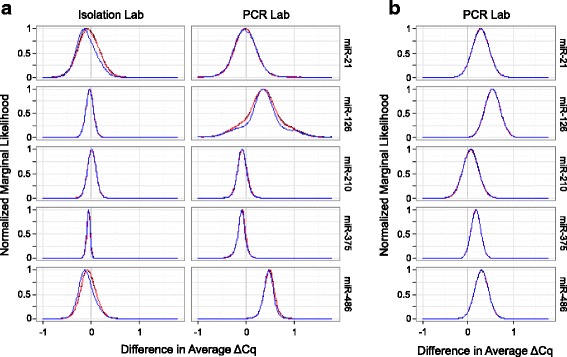
Evaluation of differences in average ΔCq (Mix1–Mix2) between measurement scenarios differing only by measurement or isolation lab. The y-axis reflects the marginal likelihood (normalized so that the maximum is one). Panel **a** data based on average Cq values inferred for mixtures 1 and 2 derived using Eq. 1 and the average Cq of component cell lines. Isolation lab effect determined by the average difference in ΔCq corresponding to samples isolated at BDL versus those isolated at BRL, for the in silico mixtures. PCR lab effect determined by the average difference in ΔCq corresponding to samples measured at BDL versus those measured at BRL, for the in silico mixtures. Panel **b** data based on mixtures of RNA isolated and prepared in the BRL. PCR lab effect determined by the average difference in ΔCq corresponding to samples measured at BDL versus those measured at BRL. Black, red, and blue lines correspond to model variations 1, 2, and 3, respectively, as described in Additional file 1

To characterize potential biases between labs, Fig. 8 portrays the marginal likelihood for the difference in average ΔCq values (Mix1 – Mix2) among six measurement scenarios that share source material but differ in either PCR lab or isolation lab, the seventh scenario using cell line mixtures was only measured by one lab (see Figs. 1 and 2 and Additional file 1:Table S2). In panel a, in silico modeling of Mix1 and Mix2 (see Methods) was used to predict ΔCq values, and the differences in the predicted ΔCq values between the BDL and BRL, either as the isolation lab or as the PCR lab, were used to assess laboratory bias for those two steps of the measurement process. These modeling efforts suggest that the likelihood of the observed data is largest under models for which biases between isolation labs are near zero, supporting a hypothesis that different labs isolating RNA from the same source can provide material with similar miRNA ratio profiles.

The likelihoods of the observed data for miR-21, miR-210, and miR-375, respectively, are largest under models for which biases due to PCR lab are near zero. However, for both miR-126 and miR-486 and for both the in silico mixtures (Fig. 8a) and the RNA mixtures (Fig. 8b), the likelihoods are largest under models for which the average ΔCq value corresponding to PCR measurements conducted at the BDL are roughly 0.5 greater than the average ΔCq value corresponding to PCR measurements conducted at the BRL. These interpretations are consistent with the spatial separation observed in Fig. 4b when comparing open symbols (BDL) and filled symbols (BRL) for miR-126 and miR-486.

If the measurements in phases 1 and 2 had been deemed insufficiently reproducible, additional measurements could be obtained to confirm the apparent biases along with efforts to identify differences in how PCR measurements were conducted at each lab for each combination of cell line and either miR-126 or miR-486. For the proof-of-concept purposes of the present study, the exhibited reproducibility was deemed adequate and no further attempts were made to investigate or control the observed variability.

Although the source material for each of these measurement scenarios was obtained from different expansions of the cell lines, the direction of fold-change between samples for each target miRNA is consistent and the order of relative abundance is the same among the miRNA targets: miRNA-486 < miRNA-375 < miRNA-126 < miRNA-210 < miR-21 (see Fig. 7).

**Fig. 9:**
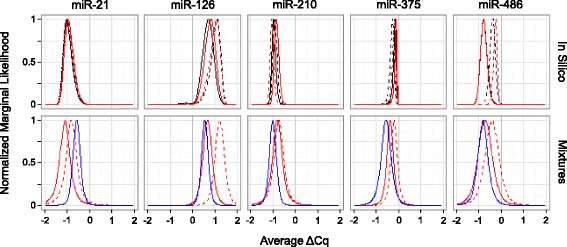
Comparison of average ΔCq (Mix1 – Mix2) across experimental workflows. The y-axis reflects the marginal likelihood (normalized so that the maximum is one). The top row of graphs compares ΔCq results from model 1 across measurement processes (see Fig. 1) using in silico modeled Cq values (see Eq. 1) for each mixture. Black and red lines correspond to samples isolated at BDL and BRL, respectively. Dashed and solid lines correspond to RT-PCR measurements from BDL and BRL, respectively. The bottom row of graphs compares ΔCq results from model 1 between mixture paradigms (see Fig. 2). Red lines correspond to RNA isolated in the BRL then mixed, blue lines correspond to RNA isolated in the BRL after mixing cells. Dashed and solid lines correspond to RT-PCR measurements from BDL and BRL, respectively

To characterize potential biases in ΔCq among source materials, Fig. 9 portrays the marginal likelihood for the average ΔCq values (Mix1 – Mix2) among the seven measurement scenarios. The top row of graphs is based on the in silico modeled ΔCq values from the cell line profiles, and the bottom row includes both the RNA mixtures and cell line mixtures. In each plot, the height of the likelihood curve reflects the chance of the observed data occurring given a model for which the true average ΔCq value is fixed at the corresponding value along the x-axis, after integrating across the modeled distribution of all other model parameters. For each miR, the peaks are either all above or all below zero, corroborating that the direction of change (negative or positive ΔCq) is consistent for each miRNA across all source material and measurement combinations. For miR-210 and miR-375, all seven peaks appear to substantially overlap meaning there is little evidence of bias among any of the measurement scenarios. For miR-126 and miR-486, there appears to be some peak separation between measurement scenarios that differ with respect to which lab conducted PCR but strong overlap among peaks corresponding to measurement scenarios that differ only by source material. The greatest variability in average ΔCq associated with source material appears to occur within miR-21, and, even then, the peaks still overlap. This statistical analysis does not conclusively indicate that all three source materials produce equivalent ΔCq values on average, nor does it conclusively refute that claim. That is, using cell lines as a regenerable source for designing cell and RNA mixture reference materials exhibits potential and warrants further study.

The reported results based on marginal likelihoods appear consistent across the three considered variations of the statistical model, indicating they are not unique to one specific subjective modeling choice. Further consideration of additional models not evaluated in this analysis may produce results that differ substantially. Analyzing additional samples would further reduce the uncertainty underlying these assessments.

To the extent that variability in average ΔCq among the source materials may be present, some of this difference might be due to drift in cell line profiles, since cells used for in silico modeling were cultured several months before those used to produce the RNA and cell mixtures and the individual cell lines were not separately profiled again at the time the mixtures were prepared. Preparing a sufficiently large “batch” of each cell line from one expansion to partition into source material for individual cell line profiles, total RNA mixtures, and cell line mixtures might reduce variability among observed ΔCq values for each analyte. In addition, determining the miRNA fraction per cell line may also allow for further refinement of the target values derived from cell line profiles and provide a more quantitative assessment of measurement performance. A difference in mRNA fraction has been shown to affect the expected log ratios in other gene expression experiments [23]. A similar use of synthetic miRNA spike-ins introduced into these RNA mixtures could help determine if there are differences in the miRNA fraction in total RNA. Further stabilizing ΔCq between replicate sample pairs may require additional optimization of cell growth conditions or identification of cell lines with greater differences among the miRNA profiles.

## Conclusions

Cell line mixtures can be prepared with designed-in differences between samples in a reproducible process and may provide suitable reference samples to assess the entire measurement process from RNA extraction through miRNA measurement. The selection of potential cell lines may be based on previous laboratory experience with material or derived from the literature (including cell repository descriptions). The screening of candidate cell lines by multiple laboratories was useful in establishing robust differences in expression for a selected set of miRNAs. In silico modeling of these levels informed the final mixture design for both RNA mixtures and mixtures of cells. Identifying cell lines with even greater differences between miRNA profiles might allow for mixtures that provide a broader range of designed-in ratios. RNA mixtures derived from these cell lines can also serve as well-matched reference samples for measurement assurance of the post-RNA isolation stages of gene expression measurement systems.

This testing paradigm should be generalizable to other biomarkers and other laboratories and provide a model for establishing both intra- and inter-laboratory measurement assurance and demonstrate translatability of biomarker assays between research development laboratories and clinical reference laboratories.

## Additional files

Additional file 1: Intro to Bayesian analysis. (PDF 1844 kb)

Additional file 2: Cell line profile Cq values. (CSV 13 kb)

Additional file 3: RNA mixture Cq values. (CSV 18 kb)

Additional file 4: Cell line mixture Cq values. (CSV 8 kb)

Additional file 5: Code for running MCMC. (PDF 41 kb)

Additional file 6: Phase1 ANOVA parsed by analyte and cell line. (PDF 78 kb)

## Abbreviations

BDL: biomarker discovery laboratory
BRL: biomarker reference laboratory
CAP: College of American Pathology
CLIA: Clinical Laboratory Improvement Amendments
EDRN: Early Detection Research Network
MCMC: Markov Chain Monte Carlo
miRNA: microRNA
NIST: National Institute of Standards and Technology
RT-PCR: reverse transcription polymerase chain reaction
