# Supplementary material for: Cell-based reference samples designed with specific differences in microRNA biomarkers
Source: BMC Biotechnol. 2018 Mar 20;18:17. doi: 10.1186/s12896-018-0423-4 (PMC5859499; doi:10.1186/s12896-018-0423-4)
Supplement: Supplementary file 1 — Intro to Bayesian analysis. (PDF 1844 kb) [file 12896_2018_423_MOESM1_ESM.pdf]

Brief Introduction to Bayesian analysis

Suppose an experiment having produced data collectively denoted by Y is to be analyzed according to a model for which the unknown components are collectively denoted by Θ, referred to as model parameters. The Bayesian framework relies upon an analyst representing their preexisting uncertainty regarding possible model parameter values as a probability distribution (called a prior distribution) and invokes Bayes theorem, which provides a mechanism for updating one's preexisting uncertainty on the basis of information gained from new data in a manner that abides the rules of probability theory. Bayes theorem dictates that the posterior distribution (or updated uncertainty) for the model parameters having value θ is proportional to the product of the prior distribution for the model parameters having value θ and the likelihood of the data according to a model with parameter values θ. That is,

$$\text{Posterior} = \text{k} \cdot \text{Prior} \cdot \text{Likelihood},$$

or, more formally,

$$Pr(\Theta = \theta|Y) = k \cdot Pr(\Theta = \theta) \cdot Pr(Y|\Theta = \theta), \qquad \text{(S1)}$$

where θ is free to assume any values the model parameters may have and k is a normalization constant that ensures the posterior distribution integrates to one. In this expression, $Prior(\Theta = \theta)$ reflects a particular analyst's preexisting belief (subjectively formed, possibly based on their previous experience or expertise) as to how probable are various values for the given model parameter. $Pr(Y|\Theta = \theta)$ in **Equation S1** may be described as the likelihood of the observed data given the model for which the parameters are equal to θ. (Note that values for all model parameters must be specified before a likelihood can be computed. If some of the model parameters are not of interest, called nuisance parameters, the likelihood in **Equation S1** can be replaced by a marginal likelihood, which is a weighted average of likelihoods obtained by using all possible combinations of nuisance parameter values. The weights used in this averaging are determined by the assumed distributions for nuisance parameters.) The likelihood reflects how data from the current experiment should influence the analyst's previous beliefs, presuming they choose to abide by the rules of probability theory. Importantly, likelihoods (marginal or not) do *not* indicate the probability of model parameters assuming a particular value, but rather how likely the data are under particular modeling assumptions.

This paragraph provides a very brief example for the benefit of interested readers. Suppose θ and θ' represent two possible values for unknown model parameters (Θ) and that the analyst provides a prior distribution for Θ such that $Prior(\Theta = \theta) = 2 \cdot Prior(\Theta = \theta')$. This would indicate that the analyst believes (before considering any data from the current experiment) that Θ is twice as probable to equal θ as it is to equal θ'. Suppose further that according to the analyst's model, the observed data is three times as likely to occur when Θ equals θ as when Θ equals θ' [i.e., $Pr(Y|\Theta = \theta) = 3 \cdot Pr(Y|\Theta = \theta')$]. Invoking Bayes theorem yields

$$\begin{aligned} Posterior(\Theta = \theta|Y) &= k \cdot Prior(\Theta = \theta) \cdot \mathrm{Pr}(Y|\Theta = \theta) \\ &= k \cdot 2 \cdot Prior(\Theta = \theta') \cdot 3 \cdot \mathrm{Pr}(Y|\Theta = \theta') \\ &= 6 \cdot Posterior(\Theta = \theta'|Y). \end{aligned}$$

Thus, after considering the new data, the analyst should come away believing that Θ is six times as probable to equal θ as it is to equal θ'.

Analysis of the Cq Data

Data collected throughout the experiments described in the main text were analyzed in a Bayesian framework to evaluate the uncertainty underlying quantities of interest related to average Cq values or their differences (e.g., average ΔCq values). In the analysis of each miR, model parameters representing the average Cq value for each of 18 distinct combinations of source material, measurement method and target are fit and subsequently used to evaluate and compare average ΔCq values (Mix1-Mix2, representing the log2 ratio between Mix1 and Mix2 abundance) for seven different measurement scenarios. The 18 combinations for which average Cq values were modeled and the seven measurement scenarios for which corresponding average ΔCq values were modeled are outlined in **Table S1** and **Table S2**, respectively.

Combinations	Source Material	Isolation Lab	PCR Lab
1 to 12	Cell H226, Cell H358, Cell H460	BDL, BRL	BDL, BRL
13 to 16	RNA Mix1, RNA Mix2	BRL	BDL, BRL
17 to 18	Cell Mix1, Cell Mix2	BRL	BRL

Table S1: Combinations of experimental factors for which average Cq values were estimated. Factorial combinations of all reported factor levels within each row were included.

Scenarios	Source Material	Isolation Lab	PCR Lab
1 to 4	In Silico Mixtures	BDL, BRL	BDL, BRL
5 to 6	RNA Mixtures	BRL	BDL, BRL
7	Cell Mixtures	BRL	BRL

Table S2: Combinations of experimental factors for which average ?Cq (Mix1-Mix2) values were estimated. Factorial combinations of all reported factor levels within each row were included.

For each target miR, the observed Cq values were analyzed using mixed effects models that include a fixed mean for each of the 18 distinct combinations of source material, measurement method and target, as well as random effects for plate and isolation. To examine the sensitivity of the results to modeling choices, the entire analysis was repeated using three model perturbations, which are described at the end of this document. In each case, the models for each analyte were fit using Markov Chain Monte Carlo (MCMC) evaluation via the R package rjags [1,2]. MCMC is a computational tool commonly used in Bayesian analysis that relies on simulation over many iterations to approximate closed-form computations that are difficult to evaluate because they involve complicated probability distributions.

At each iteration of simulation, MCMC randomly samples a value for each unknown parameter in a model according to probability distributions specified under that model. Collectively, across many iterations, the set of values ascribed to a parameter form a sample used to estimate that parameter's posterior distribution, as defined by the distributional assumptions of the model.

For MCMC iteration b in which the assigned value for the average Cq for cell line c among samples isolated at lab i and measured at PCR lab p is $\mu_{cip}^{(b)}$, the corresponding average Cq for *in silico* mixtures of Mix1 and Mix2 were approximated using the following equation:

$$\hat{\mu}_{cip}^{(b)} = -log_2 \sum_{c=1}^{3} 2^{-\mu_{cip}^{(b)}} * \phi_{cm} \qquad \text{(S2)}$$

where $\hat{\mu}_{mip}$ is an approximation to the hypothetical average Cq measurement for designed mixture m consisting of relative concentrations of ϕ_{1m}, ϕ_{2m}, and ϕ_{3m} for component cell lines H226, H358 and H460, respectively, prepared at isolation lab i and measured at PCR lab p. (This method of approximation is inexact due to Jensen's inequality, which states that the average of a non-linear function applied to a set of values is not equal to the non-linear function applied to the average of the set of values. However, the authors view this method of approximation as being fit-for-purpose.) For each of the seven measurement scenarios in **Table S2**, the average ΔCq value for each MCMC iteration was obtained by taking the difference between the corresponding Mix1 and Mix2 average Cq values. Measurement scenarios were compared (pairwise) by taking the difference between their respective average ΔCq values for each MCMC iteration. The collection of MCMC iterations thus produced a sample for the posterior distribution of the apparent bias between measurement scenarios in their log2 ratio determinations.

Equation S1 clearly conveys that the posterior distribution may be explicitly influenced by the subjective choice of prior distribution. In an effort to reduce the subjectivity in the reported results, we use the MCMC sample to approximate the marginal likelihood, rather than focus on the posterior distributions. (A posterior distribution purports to reflect the probability a model parameter has a particular value or falls within a specified interval, given the observed data. A marginal likelihood purports to reflect only how likely the observed data would be to occur, given a particular value for the parameter of interest, on average with respect to the assumed distribution of other model parameters. Likelihoods do not indicate the probability of model parameter assuming a particular value.) Specifically, for each parameter of interest, we present the ratio of the posterior distribution to the prior distribution, as approximated by kernel density estimation applied to MCMC samples for the posterior and prior distributions, respectively. To the extent that a particular observer would have viewed each possible value within a considered interval as equally probable before observing the experimental results and agrees with distributional assumptions for the other model parameters, the marginal likelihood profiles over this interval are proportional to the posterior probability density that observer would arrive at following Bayes rule.

Model Descriptions

The following model framework was used to analyze the observed Cq values (obtained as the average of triplicate measurements) for each target miR independently. The model description is given in the context of a single target miR and was independently repeated for each of the five target miRs in the experiment.

The RT-PCR measurements were analyzed using the following mixed effects linear model

$$Y_{cteipr} = \mu_{ctip} + \gamma_{tei} + \delta_{teic} + \rho_{ctp} + \varepsilon_{cteipr}, \qquad \text{(S3)}$$

where Y_{cteipr} is the recorded Cq value corresponding to measurement replicate r at PCR lab p for cell line contents c (c indicates to which of the following choices the considered Cq value pertains: component cell line H226, H358, or H460; Mix1 or Mix2) in mixture type t (indicating no mixture, RNA mixture or cell line mixture) obtained from extracted RNA replicate e at isolation lab i; μ_{ctip} is the (fixed) average RT-PCR measurement for samples containing cell lines c in mixture type t isolated at lab i and measured at lab p; γ_{tei} is a random effect associated with extraction date e at isolation lab i for mixture type t (shared across all cell lines); δ_{teic} is a random effect of extraction date e at isolation lab i for mixture type t of cell line contents c; ρ_{ctp} is a random effect of the plate shared by all measurements occurring at PCR lab p of analyte a in cell line contents c for mixture type t; and ε_{cteipr} is a random error in measurement replicate r at PCR lab p for cell line contents c in mixture type t obtained from extraction replicate e at isolation lab i. All random effects were assumed to be independent of one another with mean zero.

In the first model, all random effects were assumed to follow a normal distribution. The standard deviations of random effects γ_{tei}, δ_{teic}, ρ_{ctp}, and ε_{cteipr} are denoted as $\sigma_{\gamma t}$, $\sigma_{\delta t}$, σ_{ρ}, and σ_{ε}, respectively. That is, the standard deviations for random effects corresponding isolation date and the interaction between isolation date and cell line contents was allowed to differ independently across the mixture types. The standard deviations for random effects corresponding to plate and measurement error were fixed across the mixture types. The following vague prior distributions were placed on the model parameters: each μ_{ctip} was assigned a uniform distribution from 15 to 40; $\sigma_{\gamma t}$, $\sigma_{\delta t}$, σ_{ρ}, and σ_{ε} were assigned exponential distributions with mean 1.

The second model differed from the first only by the priors assigned to $\sigma_{\gamma t}$, $\sigma_{\delta t}$, σ_{ρ}, and σ_{ε}, which were assigned exponential distributions with mean 5, rather than mean 1.

If the third model, the prior distribution for each μ_{ctip} was normal with mean 27.5 and standard deviation 10. All random effects were assumed to follow double exponential distributions, rather than normal distributions as used in the first two models. The scale parameters for distributions of random effects $\square_{tei}$, δ_{teic}, ρ_{ctp}, and ε_{cteipr} are denoted as $\sigma_{\gamma t}$, $\sigma_{\delta t}$, σ_{ρ}, and σ_{ε}, respectively, analogous to the standard deviations used in models 1 and 2. The scale parameters were assigned exponential prior distributions with mean 1.

In each case, 250 000 MCMC iterations were executed, recording parameter values from every 25th iteration, following a burn-in of 100 000 iterations for each of 5 independent chains. Posterior distributions for quantities of interest are based on computations from each of the 50 000 sets of model parameter values, as described in the main text. For each model, prior distributions used to evaluate marginal likelihoods are based on computations from 100 000 samples from the prior distributions assigned to μ_{ctip} under the given model. The final density used for both posteriors and priors was obtained as Gaussian kernel density estimates using bandwidths obtained from the default settings of the R function *density*, applied to the values resulting from MCMC evaluation described above.

References

1. R Core Team (2014). R: A language and environment for statistical computing. R Foundation for Statistical Computing, Vienna, Austria. URL http://www.R-project.org/.
2. Martyn Plummer (2014). rjags: Bayesian graphical models using MCMC. R package version 3-14. http://CRAN.R-project.org/package=rjags.
