# Supplementary material for: Cell-based reference samples designed with specific differences in microRNA biomarkers
Source: BMC Biotechnol. 2018 Mar 20;18:17. doi: 10.1186/s12896-018-0423-4 (PMC5859499; doi:10.1186/s12896-018-0423-4)
Supplement: Supplementary file 5 — Code for running MCMC. (PDF 41 kb) [file 12896_2018_423_MOESM5_ESM.pdf]

```
setwd("C:/ ... ”) ### Replace ... with path Phase*.csv to datasets
library(ggplot2)
library(grid)
library(tidyr)
library(dplyr)
require(rjags)
require(R2WinBUGS)
require(lme4)
library(gridExtra)
library(grid)

Phase1_all <- read.csv("Phase1_Factors.csv")
### Fourth cell line excluded from analysis due to difficulties
growing in lab
Phase1_all<-Phase1_all[Phase1_all$Cell_Line!="SK-Mes1",]
Phase1_all$Plate<-
as.factor(paste(Phase1_all$Cell_Line,Phase1_all$Analyte,Phase1_all$PCR
_Lab))
Phase2_all <- read.csv("Phase2_Factors.csv")
Phase2_all$Iso_Lab <-"BRL"
Phase2_all$Isolation <-substr(Phase2_all$Sample,6,6)
Phase3_all <- read.csv("Phase3_Factors.csv")
Phase3_all$Iso_Lab <-"BRL"
Phase3_all$Isolation <-substr(Phase3_all$Sample,6,6)

### Define three different models used for MCMC analysis
model.list<-list()
### First model
model.list[[1]]<-function(){
  ## prior definitions
  for(c in 1:3){
    for(m in 1:4){
      cell.method1[c,m]~dunif(15,40)
    }
  }
  for(c in 1:2){
    for(m in 1:2){
      mix.method2[c,m]~dunif(15,40)
    }
  }
  for(c in 1:2){
    mix.method3[c]~dunif(15,40)
  }

  sd.isolation1 ~ dexp(.5)
  sd.isolation2 ~ dexp(.5)
  sd.isolation3 ~ dexp(.5)
  sd.ic1 ~ dexp(.5)
  sd.im2 ~ dexp(.5)
  sd.im3 ~ dexp(.5)
```

```
sd.plate ~ dexp(.5)
sd.error ~ dexp(.5)

tau.isolation1 <- 1/(sd.isolation1*sd.isolation1)
tau.isolation2 <- 1/(sd.isolation2*sd.isolation2)
tau.isolation3 <- 1/(sd.isolation3*sd.isolation3)
tau.ic1 <- 1/(sd.ic1*sd.ic1)
tau.im2 <- 1/(sd.im2*sd.im2)
tau.im3 <- 1/(sd.im3*sd.im3)
tau.plate <- 1/(sd.plate*sd.plate)
tau.error <- 1/(sd.error*sd.error)
#Random effects

for(i in 1:6){
  isolation1[i] ~ dnorm(0,tau.isolation1)
  for(c in 1:3){
    ic1[i,c] ~ dnorm(0,tau.ic1)
  }
}

for(i in 1:4){
  isolation2[i] ~ dnorm(0,tau.isolation2)
  isolation3[i] ~ dnorm(0,tau.isolation3)
  for(m in 1:2){
    im2[i,m]~ dnorm(0,tau.im2)
    im3[i,m]~ dnorm(0,tau.im3)
  }
}

for(i in 1:6){
  plate1[i] ~ dnorm(0, tau.plate)
  plate2[i] ~ dnorm(0, tau.plate)
}
for(i in 1:3){
  plate3[i] ~ dnorm(0, tau.plate)
}

#Model for data
for(i in 1:n1_obs){
  mn1[i]<-cell.method1[c1.ind[i],m1.ind[i]]+isolation1[i1.ind[i]]+
    plate1[p1.ind[i]]+ic1[i1.ind[i],c1.ind[i]]
  Ct1[i]~dnorm(mn1[i],tau.error)
}

for(i in 1:n2_obs){
  mn2[i]<-mix.method2[mix2.ind[i],m2.ind[i]]+isolation2[i2.ind[i]]+
    plate2[p2.ind[i]]+im2[i2.ind[i],mix2.ind[i]]
  Ct2[i]~dnorm(mn2[i],tau.error)
}
```

```
  for(i in 1:n3_obs){
    mn3[i]<-mix.method3[mix3.ind[i]]+isolation3[i3.ind[i]]+
      plate3[p3.ind[i]]+im3[i3.ind[i],mix3.ind[i]]
    Ct3[i]~dnorm(mn3[i],tau.error)
  }
}
### Second model
model.list[[2]]<-function(){
  ## prior definitions
  for(c in 1:3){
    for(m in 1:4){
      cell.method1[c,m]~dunif(15,40)
    }
  }
  for(c in 1:2){
    for(m in 1:2){
      mix.method2[c,m]~dunif(15,40)
    }
  }
  for(c in 1:2){
    mix.method3[c]~dunif(15,40)
  }

  sd.isolation1 ~ dexp(1)
  sd.isolation2 ~ dexp(1)
  sd.isolation3 ~ dexp(1)
  sd.ic1 ~ dexp(1)
  sd.im2 ~ dexp(1)
  sd.im3 ~ dexp(1)
  sd.plate ~ dexp(1)
  sd.error ~ dexp(1)

  tau.isolation1 <- 1/(sd.isolation1*sd.isolation1)
  tau.isolation2 <- 1/(sd.isolation2*sd.isolation2)
  tau.isolation3 <- 1/(sd.isolation3*sd.isolation3)
  tau.ic1 <- 1/(sd.ic1*sd.ic1)
  tau.im2 <- 1/(sd.im2*sd.im2)
  tau.im3 <- 1/(sd.im3*sd.im3)
  tau.plate <- 1/(sd.plate*sd.plate)
  tau.error <- 1/(sd.error*sd.error)
  #Random effects

  for(i in 1:6){
    isolation1[i] ~ dnorm(0,tau.isolation1)
    for(c in 1:3){
      ic1[i,c] ~ dnorm(0,tau.ic1)
    }
  }

  for(i in 1:4){
```

```
    isolation2[i] ~ dnorm(0,tau.isolation2)
    isolation3[i] ~ dnorm(0,tau.isolation3)
    for(m in 1:2){
      im2[i,m]~ dnorm(0,tau.im2)
      im3[i,m]~ dnorm(0,tau.im3)
    }
  }

  for(i in 1:6){
    plate1[i] ~ dnorm(0, tau.plate)
    plate2[i] ~ dnorm(0, tau.plate)
  }
  for(i in 1:3){
    plate3[i] ~ dnorm(0, tau.plate)
  }

  #Model for data
  for(i in 1:n1_obs){
    mn1[i]<-cell.method1[c1.ind[i],m1.ind[i]]+isolation1[i1.ind[i]]+
      plate1[p1.ind[i]]+ic1[i1.ind[i],c1.ind[i]]
    Ct1[i]~dnorm(mn1[i],tau.error)
  }

  for(i in 1:n2_obs){
    mn2[i]<-mix.method2[mix2.ind[i],m2.ind[i]]+isolation2[i2.ind[i]]+
      plate2[p2.ind[i]]+im2[i2.ind[i],mix2.ind[i]]
    Ct2[i]~dnorm(mn2[i],tau.error)
  }

  for(i in 1:n3_obs){
    mn3[i]<-mix.method3[mix3.ind[i]]+isolation3[i3.ind[i]]+
      plate3[p3.ind[i]]+im3[i3.ind[i],mix3.ind[i]]
    Ct3[i]~dnorm(mn3[i],tau.error)
  }
}
### Third model
model.list[[3]]<-function(){
  ## prior definitions
  for(c in 1:3){
    for(m in 1:4){
      cell.method1[c,m]~dnorm(27.5,.01)
    }
  }
  for(c in 1:2){
    for(m in 1:2){
      mix.method2[c,m]~dnorm(27.5,.01)
    }
  }
  for(c in 1:2){
    mix.method3[c]~dnorm(27.5,.01)
```

```
}

sd.isolation1 ~ dexp(1)
sd.isolation2 ~ dexp(1)
sd.isolation3 ~ dexp(1)
sd.ic1 ~ dexp(1)
sd.im2 ~ dexp(1)
sd.im3 ~ dexp(1)
sd.plate ~ dexp(1)
sd.error ~ dexp(1)

tau.isolation1 <- 1/(sd.isolation1*sd.isolation1)
tau.isolation2 <- 1/(sd.isolation2*sd.isolation2)
tau.isolation3 <- 1/(sd.isolation3*sd.isolation3)
tau.ic1 <- 1/(sd.ic1*sd.ic1)
tau.im2 <- 1/(sd.im2*sd.im2)
tau.im3 <- 1/(sd.im3*sd.im3)
tau.plate <- 1/(sd.plate*sd.plate)
tau.error <- 1/(sd.error*sd.error)
#Random effects

for(i in 1:6){
  isolation1[i] ~ ddexp(0,tau.isolation1)
  for(c in 1:3){
    ic1[i,c] ~ ddexp(0,tau.ic1)
  }
}

for(i in 1:4){
  isolation2[i] ~ ddexp(0,tau.isolation2)
  isolation3[i] ~ ddexp(0,tau.isolation3)
  for(m in 1:2){
    im2[i,m]~ ddexp(0,tau.im2)
    im3[i,m]~ ddexp(0,tau.im3)
  }
}

for(i in 1:6){
  plate1[i] ~ ddexp(0, tau.plate)
  plate2[i] ~ ddexp(0, tau.plate)
}
for(i in 1:3){
  plate3[i] ~ ddexp(0, tau.plate)
}

#Model for data
for(i in 1:n1_obs){
  mn1[i]<-cell.method1[c1.ind[i],m1.ind[i]]+isolation1[i1.ind[i]]+
    plate1[p1.ind[i]]+ic1[i1.ind[i],c1.ind[i]]
  Ct1[i]~dnorm(mn1[i],tau.error)
```

```
  }

  for(i in 1:n2_obs){
    mn2[i]<-mix.method2[mix2.ind[i],m2.ind[i]]+isolation2[i2.ind[i]]+
      plate2[p2.ind[i]]+im2[i2.ind[i],mix2.ind[i]]
    Ct2[i]~dnorm(mn2[i],tau.error)
  }

  for(i in 1:n3_obs){
    mn3[i]<-mix.method3[mix3.ind[i]]+isolation3[i3.ind[i]]+
      plate3[p3.ind[i]]+im3[i3.ind[i],mix3.ind[i]]
    Ct3[i]~dnorm(mn3[i],tau.error)
  }
}


### Run MCMC for each of the 3 different models
for(model in 1:3){
  Ct_model<-model.list[[model]]
  filename="Ct_model.bug"
  write.model(Ct_model, filename)
  samps<-list()
  ###Analyze each miR separately
  for(an.ind in 1:5){
    ## Which miR is currently being analyzed?
    an<-unique(Phase1_all$Analyte)[an.ind]

    ### Get subset of data that pertains to current miR
    t.Phase1<-Phase1_all[Phase1_all$Analyte==an,]
    t.Phase2<-Phase2_all[Phase2_all$Analyte==an,]
    t.Phase3<-Phase3_all[Phase3_all$Analyte==an,]

    ### Define indices as used in model definition
    c1.ind<-as.numeric(as.factor(t.Phase1$Cell_Line))
    i1.ind<-
as.numeric(as.factor(paste(t.Phase1$Iso_Lab,t.Phase1$Isolation)))
    m1.ind<-
as.numeric(as.factor(paste(t.Phase1$PCR_Lab,t.Phase1$Iso_Lab)))
    p1.ind<-
as.numeric(as.factor(paste(t.Phase1$PCR_Lab,t.Phase1$Plate)))
    n1_obs<-nrow(t.Phase1)

    mix2.ind<-as.numeric(as.factor(t.Phase2$Mixture))
    i2.ind<-as.numeric(as.factor(t.Phase2$Isolation))
    m2.ind<-as.numeric(as.factor(t.Phase2$PCR_Lab))
    p2.ind<-
as.numeric(as.factor(paste(t.Phase2$PCR_Lab,t.Phase2$Plate)))

    mix3.ind<-as.numeric(as.factor(t.Phase3$Mixture))
    i3.ind<-as.numeric(as.factor(t.Phase3$Isolation))
```

```
    p3.ind<-as.numeric(as.factor(t.Phase3$Plate))

    ## Run MCMC using jags
    mod<-jags.model(file=filename,list( c1.ind= c1.ind,
                                        i1.ind=i1.ind,
                                        m1.ind= m1.ind,
                                        p1.ind= p1.ind,
                                        n1_obs= nrow(t.Phase1),
                                        Ct1 = t.Phase1$Analyte_Ct,
                                        mix2.ind= mix2.ind,
                                        i2.ind=i2.ind,
                                        m2.ind= m2.ind,
                                        p2.ind= p2.ind,
                                        n2_obs= nrow(t.Phase2),
                                        Ct2 = t.Phase2$Analyte_Ct,
                                        mix3.ind= mix3.ind,
                                        i3.ind=i3.ind,
                                        p3.ind= p3.ind,
                                        n3_obs= nrow(t.Phase3),
                                        Ct3 = t.Phase3$Analyte_Ct),
                    inits=NULL, n.chains = 5, n.adapt=10000,
quiet=FALSE)
    update(object=mod, n.iter=100000)#, by, progress.bar

    ### Store parameter values obtained during MCMC simulation
    samps<-jags.samples(model=mod,
                                  variable.names=c("cell.method1",
                                                   "mix.method2",
                                                   "mix.method3",
                                                   "sd.isolation1",
                                                   "sd.isolation2",
                                                   "sd.isolation3",
                                                   "sd.ic1",
                                                   "sd.im2",
                                                   "sd.im3",
                                                   "sd.plate",
                                                   "sd.error"),
                                  n.iter=250000, thin = 25,
type="trace")
  }
  ## Save MCMC results
  #save(samps,file=paste0("MCMC_Results_from_Model",model,".Rdata"))
}


##############################################################
### Code to generate Figure 4 from MCMC simulation results ###
##############################################################

xx<-seq(-1.95,1.95,by=.01)
```

```
prior<-matrix(NA,length(xx),7)
diff.res<-NULL
for(model in 1:3){
  load(file=paste0("MCMC_Results_from_Model",model,".Rdata"))

  for(an.ind in 1:5){
    an<-unique(Phase1_all$Analyte)[an.ind]
    print(paste0("Model ",model,"; Analyte ",an))

    mn1<-summary(samps[[an.ind]]$cell.method1,FUN=function(x)x)$stat

    ###Mix 1 cell line proportions
    M1H460 <- 0.20
    M1H358 <- 0.60
    M1H226 <- 0.20
    ###Mix 2 cell line proportions
    M2H460 <- 0.20
    M2H358 <- 0.20
    M2H226 <- 0.60

    Mix1<-Mix2<-matrix(NA,dim(mn1)[1],4)
    for(m in 1:4){
      Mix1[,m]<- -log2(((2^-mn1[,3,m])*M1H460) +
                       ((2^-mn1[,2,m])*M1H358) +
                       ((2^-mn1[,1,m])*M1H226))
      Mix2[,m]<- -log2(((2^-mn1[,3,m])*M2H460) +
                       ((2^-mn1[,2,m])*M2H358) +
                       ((2^-mn1[,1,m])*M2H226))
    }

    dCt1<-Mix1-Mix2
    colnames(dCt1)<-c("BDL BDL","BDL BRL","BRL BDL","BRL BRL")

    mn2<-summary(samps[[an.ind]]$mix.method2,FUN=function(x)x)$stat
    dCt2<-mn2[,1,]-mn2[,2,]
    colnames(dCt2)<-c("BDL BRL","BRL BRL")

    mn3<-summary(samps[[an.ind]]$mix.method3,FUN=function(x)x)$stat
    dCt3<-mn3[,1]-mn3[,2]

    if(model<3){ ### What do priors for differences in delta Ct look
like under models 1 and 2?
      N<-1e5
      prior.Mix1<-prior.Mix2<-matrix(NA,N,4)
      for(m in 1:4){
        H460<-runif(N,15,40)
        H358<-runif(N,15,40)
        H226<-runif(N,15,40)
        prior.Mix1[,m]<- -log2(((2^-H460)*M1H460) +
                                ((2^-H358)*M1H358) +
```

```
                              ((2^-H226)*M1H226))
          prior.Mix2[,m]<- -log2(((2^-H460)*M2H460) +
                              ((2^-H358)*M2H358) +
                              ((2^-H226)*M2H226))
        }

        prior.dCt1<-prior.Mix1-prior.Mix2
        prior.dCt2<-cbind(runif(N,15,40)-runif(N,15,40),runif(N,15,40)-
runif(N,15,40))
        prior.dCt3<-runif(N,15,40)-runif(N,15,40)
      }

      if(model==3){ ### What do priors for differences in delta Ct look
like under model 3?
        N<-1e5
        prior.Mix1<-prior.Mix2<-matrix(NA,N,4)
        for(m in 1:4){
          H460<-rnorm(N,27.5,10)
          H358<-rnorm(N,27.5,10)
          H226<-rnorm(N,27.5,10)
          prior.Mix1[,m]<- -log2(((2^-H460)*M1H460) +
                              ((2^-H358)*M1H358) +
                              ((2^-H226)*M1H226))
          prior.Mix2[,m]<- -log2(((2^-H460)*M2H460) +
                              ((2^-H358)*M2H358) +
                              ((2^-H226)*M2H226))
        }

        prior.dCt1<-prior.Mix1-prior.Mix2
        prior.dCt2<-cbind(rnorm(N,27.5,10)-rnorm(N,27.5,10),rnorm(N,
27.5,10)-rnorm(N,27.5,10))
        prior.dCt3<-rnorm(N,27.5,10)-rnorm(N,27.5,10)
      }

      ### Code for differences in average delta Ct values
      for(comp in 1:3){
        if(comp==1){       #RNA BDL vs. BRL)
          samp<-dCt2[,1]-dCt2[,2]
          samp2<-prior.dCt2[,1]-prior.dCt2[,2]
        }
        if(comp==2){       #Sil PCR BDL vs. BRL)
          samp<-rowMeans(dCt1[,1:2]-dCt1[,3:4])
          samp2<-rowMeans(prior.dCt1[,1:2]-prior.dCt1[,3:4])
        }
        if(comp==3){       #Sil Isol BDL vs. BRL)
          samp<-rowMeans(dCt1[,c(1,3)]-dCt1[,c(2,4)])
          samp2<-rowMeans(prior.dCt1[,c(1,3)]-prior.dCt1[,c(2,4)])
        }

        ### Kernel density estimate for posterior of delta Ct difference
```

```
      post.bw<-density(samp)$bw
      diff<-matrix(xx,length(xx),length(samp),byrow=FALSE)-
        matrix(samp,length(xx),length(samp),byrow=TRUE)
      post<-rowMeans(dnorm(diff,0,post.bw))

      ### Kernel density estimate for prior of delta Ct difference
      if(is.na(prior[1,comp])){
        prior.bw<-density(samp2)$bw
        diff2<-matrix(xx,length(xx),length(samp2),byrow=FALSE)-
          matrix(samp2,length(xx),length(samp2),byrow=TRUE)
        prior[,comp]<-rowMeans(dnorm(diff2,0,prior.bw))
      }

      t.res<-data.frame(xx=xx,yy=post/
prior[,comp],comp=comp,an=as.character(an),model=model)
      diff.res<-rbind(diff.res,t.res)
    }
  }
  if(model==2) prior[]<-NA
}

### Normalize marginal likelihood to have a maximum of 1
diff.res<-diff.res%>%group_by(model,an,comp)%>%mutate(norm=yy/max(yy))

### Set up plotting parameters
diff.res$model<-as.factor(diff.res$model)
x.labels<-c("PCR Lab",
            "PCR Lab",
            "Isolation Lab")
diff.res$comp.name<-x.labels[diff.res$comp]

### Plot marginal likelihoods for differences in delta Ct
p1<-ggplot(diff.res[diff.res$comp!=1,],aes(x=xx,y=norm,color=model)) +
  facet_grid(an~comp.name)+
  geom_segment(x=0,xend=0,y=0,yend=100,color="grey")+
  geom_path()+
  theme(axis.text=element_text(size=12),
        axis.title=element_text(size=14,face="bold"),
        strip.text=element_text(size=12))+
  xlim(c(-1,1.8))+
  xlab(bquote("Difference in Average "*Delta*"Cq"))+
  ylab("Normalized Marginal Likelihood")+
  scale_color_manual(values=c("1"="black","2"="red","3"="blue"))+
  theme_bw() +
  scale_y_continuous(breaks=(0:4)/4,labels=c(0,"","0.5","","1"))+
  theme(legend.position="none") +
  theme(strip.background = element_blank())
print(p1)

### Plot marginal likelihoods for differences in delta Ct
```

```
p2<-ggplot(diff.res[diff.res$comp==1,],aes(x=xx,y=norm,color=model)) +
  facet_grid(an~comp.name)+
  geom_segment(x=0,xend=0,y=0,yend=100,color="grey")+
  geom_path()+
  theme(axis.text=element_text(size=12),
        axis.title=element_text(size=14,face="bold"),
        strip.text=element_text(size=12))+
  xlim(c(-1,1.8))+
  xlab(bquote("Difference in Average "*Delta*"Cq"))+
  ylab("Normalized Marginal Likelihood")+
  scale_color_manual(values=c("1"="black","2"="red","3"="blue"))+
  theme_bw() +
  scale_y_continuous(breaks=(0:4)/4,labels=c(0,"","0.5","","1"))+
  theme(legend.position="none") +
  theme(strip.background = element_blank())
print(p2)
#################################
### End of Code for Figure 4  ###
#################################




##############################################################
### Code to generate Figure 6 from MCMC simulation results ###
##############################################################

### Considered range of parameter values for
### delta Cq under each of the 7 measurement processes
xx<-seq(-1.95,1.95,by=.01)
prior<-matrix(NA,length(xx),7)
res<-NULL
for(model in 1:3){
  load(file=paste0("MCMC_Results_from_Model",model,".Rdata"))

  for(an.ind in 1:5){
    an<-unique(Phase1_all$Analyte)[an.ind]
    print(paste0("Model ",model,"; Analyte ",an))

    ### Retrieve MCMC estimates of average Ct for each component
    ### cell line for each measurement method
    mn1<-summary(samps[[an.ind]]$cell.method1,FUN=function(x)x)$stat

    ###Mix 1 cell line proportions
    M1H460 <- 0.20
    M1H358 <- 0.60
    M1H226 <- 0.20
    ###Mix 2 cell line proportions
    M2H460 <- 0.20
    M2H358 <- 0.20
```

```
    M2H226 <- 0.60

    Mix1<-Mix2<-matrix(NA,dim(mn1)[1],4)
    for(m in 1:4){
      Mix1[,m]<- -log2(((2^-mn1[,3,m])*M1H460) +
                       ((2^-mn1[,2,m])*M1H358) +
                       ((2^-mn1[,1,m])*M1H226))
      Mix2[,m]<- -log2(((2^-mn1[,3,m])*M2H460) +
                       ((2^-mn1[,2,m])*M2H358) +
                       ((2^-mn1[,1,m])*M2H226))
    }

    dCt1<-Mix1-Mix2
    colnames(dCt1)<-c("BDL BDL","BDL BRL","BRL BDL","BRL BRL")

    ### Retrieve MCMC estimates of average Ct for RNA sample mix1
    ###  and mix2 under each measurement method
    mn2<-summary(samps[[an.ind]]$mix.method2,FUN=function(x)x)$stat
    dCt2<-mn2[,1,]-mn2[,2,]
    colnames(dCt2)<-c("BDL BRL","BRL BRL")

    ### Retrieve MCMC estimates of average Ct for Cell sample mix1
    ###  and mix2 under each measurement method
    mn3<-summary(samps[[an.ind]]$mix.method3,FUN=function(x)x)$stat
    dCt3<-mn3[,1]-mn3[,2]

    if(model<3){ ### What do priors for delta Ct look like under
models 1 and 2?
      N<-1e5
      prior.Mix1<-prior.Mix2<-matrix(NA,N,4)
      for(m in 1:4){
        H460<-runif(N,15,40)
        H358<-runif(N,15,40)
        H226<-runif(N,15,40)
        prior.Mix1[,m]<- -log2(((2^-H460)*M1H460) +
                               ((2^-H358)*M1H358) +
                               ((2^-H226)*M1H226))
        prior.Mix2[,m]<- -log2(((2^-H460)*M2H460) +
                               ((2^-H358)*M2H358) +
                               ((2^-H226)*M2H226))
      }

      prior.dCt1<-prior.Mix1-prior.Mix2
      prior.dCt2<-cbind(runif(N,15,40)-runif(N,15,40),runif(N,15,40)-
runif(N,15,40))
      prior.dCt3<-runif(N,15,40)-runif(N,15,40)
    }

    if(model==3){ ### What do priors for delta Ct look like under
model 3?
```

```
    N<-1e5
    prior.Mix1<-prior.Mix2<-matrix(NA,N,4)
    for(m in 1:4){
      H460<-rnorm(N,27.5,10)
      H358<-rnorm(N,27.5,10)
      H226<-rnorm(N,27.5,10)
      prior.Mix1[,m]<- -log2(((2^-H460)*M1H460) +
                             ((2^-H358)*M1H358) +
                             ((2^-H226)*M1H226))
      prior.Mix2[,m]<- -log2(((2^-H460)*M2H460) +
                             ((2^-H358)*M2H358) +
                             ((2^-H226)*M2H226))
    }

    prior.dCt1<-prior.Mix1-prior.Mix2
    prior.dCt2<-cbind(rnorm(N,27.5,10)-rnorm(N,27.5,10),rnorm(N,
27.5,10)-rnorm(N,27.5,10))
    prior.dCt3<-rnorm(N,27.5,10)-rnorm(N,27.5,10)
  }

  ### Evaluate marginal likelihoods for each delta Ct
  for(comp in 1:7){
    if(comp==1){    #cell
      material<-"Cell"
      iso.lab<-"BRL"
      pcr.lab<-"BRL"
      samp<-dCt3
      samp2<-prior.dCt3
    }
    if(comp==2){     #RNA BDL
      material<-"RNA"
      iso.lab<-"BRL"
      pcr.lab<-"BDL"
      samp<-dCt2[,1]
      samp2<-prior.dCt2[,1]
    }
    if(comp==3){       #RNA BDL
      material<-"RNA"
      iso.lab<-"BRL"
      pcr.lab<-"BRL"
      samp<-dCt2[,2]
      samp2<-prior.dCt2[,1]
    }
    if(comp==4){       #RNA.vs.sil (BDL)
      material<-"In Silico"
      iso.lab<-"BDL"
      pcr.lab<-"BDL"
      samp<-dCt1[,1]
      samp2<-prior.dCt1[,1]
    }
```

```
      if(comp==5){
        material<-"In Silico"
        iso.lab<-"BRL"
        pcr.lab<-"BDL"
        samp<-dCt1[,2]
        samp2<-prior.dCt1[,1]
      }
      if(comp==6){
        material<-"In Silico"
        iso.lab<-"BDL"
        pcr.lab<-"BRL"
        samp<-dCt1[,3]
        samp2<-prior.dCt1[,1]
      }
      if(comp==7){
        material<-"In Silico"
        iso.lab<-"BRL"
        pcr.lab<-"BRL"
        samp<-dCt1[,4]
        samp2<-prior.dCt1[,1]
      }
      ### Kernel density estimate for posterior of delta Ct
      post.bw<-density(samp)$bw
      diff<-matrix(xx,length(xx),length(samp),byrow=FALSE)-
        matrix(samp,length(xx),length(samp),byrow=TRUE)
      post<-rowMeans(dnorm(diff,0,post.bw))

      ### Kernel density estimate for prior of delta Ct
      if(is.na(prior[1,comp])){
        prior.bw<-density(samp2)$bw
        diff2<-matrix(xx,length(xx),length(samp2),byrow=FALSE)-
          matrix(samp2,length(xx),length(samp2),byrow=TRUE)
        prior[,comp]<-rowMeans(dnorm(diff2,0,prior.bw))
      }
      ## yy provides estimated normalized marginal likelihood
      ## corresponding to parameter values xx
      t.res<-data.frame(xx=xx,yy=post/prior[,comp],
                        material=material,iso.lab=iso.lab,
                        pcr.lab=pcr.lab,comp=comp,
                        an=as.character(an),
                        model=model)
      res<-rbind(res,t.res)
    }
  }

  if(model==2) prior[]<-NA
}
save(res,file="Delta_Ct_Marg_Like_Results.Rdata")
#load(file="Delta_Ct_Marg_Like_Results.Rdata")
### Normalize marginal likelihood to have a maximum of 1
```

```
res<-res%>%group_by(model,an,comp)%>%mutate(norm=yy/max(yy))

### Set up plotting parameters
line.type <- c("BDL"=2,"BRL"=1)
group.colors <- c("g1"="black","g2"="red","g3"="blue")
res$materials<-factor(as.character(res$material),
                      levels=c("In Silico","RNA","Cell"),
                      labels=c("In Silico","RNA","Cell"))
res$row<-NA
res$row[res$materials=="In Silico"]<-"In Silico"
res$row[res$materials!="In Silico"]<-"Mixtures"
res$color<-NA
res$color[res$iso.lab=="BDL"]<-"g1"
res$color[res$iso.lab=="BRL"]<-"g2"
res$color[res$materials=="Cell"]<-"g3"

### Plot marginal likelihoods for delta Ct  (model 1 only)
p3<-
ggplot(res[res$model==1,],aes(x=xx,y=norm,color=color,linetype=pcr.lab
)) +
  facet_grid(row~an)+
  geom_segment(x=0,xend=0,y=0,yend=100,color="grey",linetype=1)+
  geom_path()+
  theme(axis.text=element_text(size=12),
        axis.title=element_text(size=14,face="bold"),
        strip.text=element_text(size=12))+
  xlab(bquote("Average "*Delta*"Cq (Mix1 - Mix2)"))+
  ylab("Normalized Marginal Likelihood")+
  scale_color_manual(values=group.colors)+
  scale_linetype_manual(values=line.type)+
  scale_y_continuous(breaks=(0:4)/4,labels=c(0,"","0.5","","1"))+
  theme_bw() +
  theme(legend.position="none") +
  theme(strip.background = element_blank())
print(p3)

#### For plotting difference in average Ct values between source
materials
#### sharing common measurement process
xx<-seq(-1.95,1.95,by=.01)
prior<-matrix(NA,length(xx),7)
diff.res<-NULL
for(model in 1:3){
  load(file=paste0("MCMC_Results_from_Model",model,".Rdata"))

  for(an.ind in 1:5){
    an<-unique(Phase1_all$Analyte)[an.ind]
    print(paste0("Model ",model,"; Analyte ",an))

    mn1<-summary(samps[[an.ind]]$cell.method1,FUN=function(x)x)$stat
```

```
    ###Mix 1 cell line proportions
    M1H460 <- 0.20
    M1H358 <- 0.60
    M1H226 <- 0.20
    ###Mix 2 cell line proportions
    M2H460 <- 0.20
    M2H358 <- 0.20
    M2H226 <- 0.60

    Mix1<-Mix2<-matrix(NA,dim(mn1)[1],4)
    for(m in 1:4){
      Mix1[,m]<- -log2(((2^-mn1[,3,m])*M1H460) +
                       ((2^-mn1[,2,m])*M1H358) +
                       ((2^-mn1[,1,m])*M1H226))
      Mix2[,m]<- -log2(((2^-mn1[,3,m])*M2H460) +
                       ((2^-mn1[,2,m])*M2H358) +
                       ((2^-mn1[,1,m])*M2H226))
    }

    dCt1<-Mix1-Mix2
    colnames(dCt1)<-c("BDL BDL","BDL BRL","BRL BDL","BRL BRL")

    mn2<-summary(samps[[an.ind]]$mix.method2,FUN=function(x)x)$stat
    dCt2<-mn2[,1,]-mn2[,2,]
    colnames(dCt2)<-c("BDL BRL","BRL BRL")

    mn3<-summary(samps[[an.ind]]$mix.method3,FUN=function(x)x)$stat
    dCt3<-mn3[,1]-mn3[,2]

    if(model<3){ ### What do priors for differences in delta Ct look
like under models 1 and 2?
      N<-1e5
      prior.Mix1<-prior.Mix2<-matrix(NA,N,4)
      for(m in 1:4){
        H460<-runif(N,15,40)
        H358<-runif(N,15,40)
        H226<-runif(N,15,40)
        prior.Mix1[,m]<- -log2(((2^-H460)*M1H460) +
                               ((2^-H358)*M1H358) +
                               ((2^-H226)*M1H226))
        prior.Mix2[,m]<- -log2(((2^-H460)*M2H460) +
                               ((2^-H358)*M2H358) +
                               ((2^-H226)*M2H226))
      }

      prior.dCt1<-prior.Mix1-prior.Mix2
      prior.dCt2<-cbind(runif(N,15,40)-runif(N,15,40),runif(N,15,40)-
runif(N,15,40))
      prior.dCt3<-runif(N,15,40)-runif(N,15,40)
```

```
    }

    if(model==3){ ### What do priors for differences in delta Ct look
like under model 3?
      N<-1e5
      prior.Mix1<-prior.Mix2<-matrix(NA,N,4)
      for(m in 1:4){
        H460<-rnorm(N,27.5,10)
        H358<-rnorm(N,27.5,10)
        H226<-rnorm(N,27.5,10)
        prior.Mix1[,m]<- -log2(((2^-H460)*M1H460) +
                                 ((2^-H358)*M1H358) +
                                 ((2^-H226)*M1H226))
        prior.Mix2[,m]<- -log2(((2^-H460)*M2H460) +
                                 ((2^-H358)*M2H358) +
                                 ((2^-H226)*M2H226))
      }

      prior.dCt1<-prior.Mix1-prior.Mix2
      prior.dCt2<-cbind(rnorm(N,27.5,10)-rnorm(N,27.5,10),rnorm(N,
27.5,10)-rnorm(N,27.5,10))
      prior.dCt3<-rnorm(N,27.5,10)-rnorm(N,27.5,10)
    }

    ### Code for differences in average delta Ct values
    for(comp in 1:4){
      if(comp==1){    #cell.vs.sil
        samp<-dCt3-dCt1[,4]
        samp2<-prior.dCt3-prior.dCt1[,1]
      }
      if(comp==2){     #cell.vs.RNA
        samp<-dCt3-dCt2[,2]
        samp2<-prior.dCt3-prior.dCt1[,1]
      }
      if(comp==3){       #RNA.vs.sil (BRL)
        samp<-dCt2[,2]-dCt1[,4]
        samp2<-prior.dCt2[,1]-prior.dCt1[,1]
      }
      if(comp==4){       #RNA.vs.sil (BDL)
        samp<-dCt2[,1]-dCt1[,2]
        samp2<-prior.dCt2[,1]-prior.dCt1[,1]
      }

      ### Kernel density estimate for posterior of delta Ct difference
      post.bw<-density(samp)$bw
      diff<-matrix(xx,length(xx),length(samp),byrow=FALSE)-
        matrix(samp,length(xx),length(samp),byrow=TRUE)
      post<-rowMeans(dnorm(diff,0,post.bw))

      ### Kernel density estimate for prior of delta Ct difference
```

```
      if(is.na(prior[1,comp])){
        prior.bw<-density(samp2)$bw
        diff2<-matrix(xx,length(xx),length(samp2),byrow=FALSE)-
          matrix(samp2,length(xx),length(samp2),byrow=TRUE)
        prior[,comp]<-rowMeans(dnorm(diff2,0,prior.bw))
      }

      t.res<-data.frame(xx=xx,yy=post/
prior[,comp],comp=comp,an=as.character(an),model=model)
      diff.res<-rbind(diff.res,t.res)
    }
  }
  if(model==2) prior[]<-NA
}
save(diff.res,file="Delta_Ct_Difference_Marg_Like_Results.Rdata")
#load(file="Delta_Ct_Difference_Marg_Like_Results.Rdata")


### Normalize marginal likelihood to have a maximum of 1
diff.res<-diff.res%>%group_by(model,an,comp)%>%mutate(norm=yy/max(yy))

### Set up plotting parameters
diff.res$model<-as.factor(diff.res$model)
x.labels<-c("Cell - Sil.",
            "Cell - RNA",
            "RNA - Sil. (BDL)",
            "RNA - Sil. (BRL)")
diff.res$comp.name<-x.labels[diff.res$comp]

### Plot marginal likelihoods for differences in delta Ct
p4<-ggplot(diff.res,aes(x=xx,y=norm,color=model)) +
  facet_grid(an~comp.name)+
  geom_segment(x=0,xend=0,y=0,yend=100,color="grey")+
  geom_path()+
  theme(axis.text=element_text(size=12),
        axis.title=element_text(size=14,face="bold"),
        strip.text=element_text(size=12))+
  xlab(bquote("Difference in Average "*Delta*"Cq"))+
  ylab("Normalized Marginal Likelihood")+
  scale_color_manual(values=c("1"="black","2"="red","3"="blue"))+
  theme_bw() +
  scale_y_continuous(breaks=(0:4)/4,labels=c(0,"","0.5","","1"))+
  theme(legend.position="none") +
  theme(strip.background = element_blank())
print(p4)

#################################
### End of Code for Figure 6  ###
#################################
```
