# Supplementary material for: Cell-based reference samples designed with specific differences in microRNA biomarkers
Source: BMC Biotechnol. 2018 Mar 20;18:17. doi: 10.1186/s12896-018-0423-4 (PMC5859499; doi:10.1186/s12896-018-0423-4)
Supplement: Supplementary file 6 — Phase1 ANOVA parsed by analyte and cell line. (PDF 78 kb) [file 12896_2018_423_MOESM6_ESM.pdf]

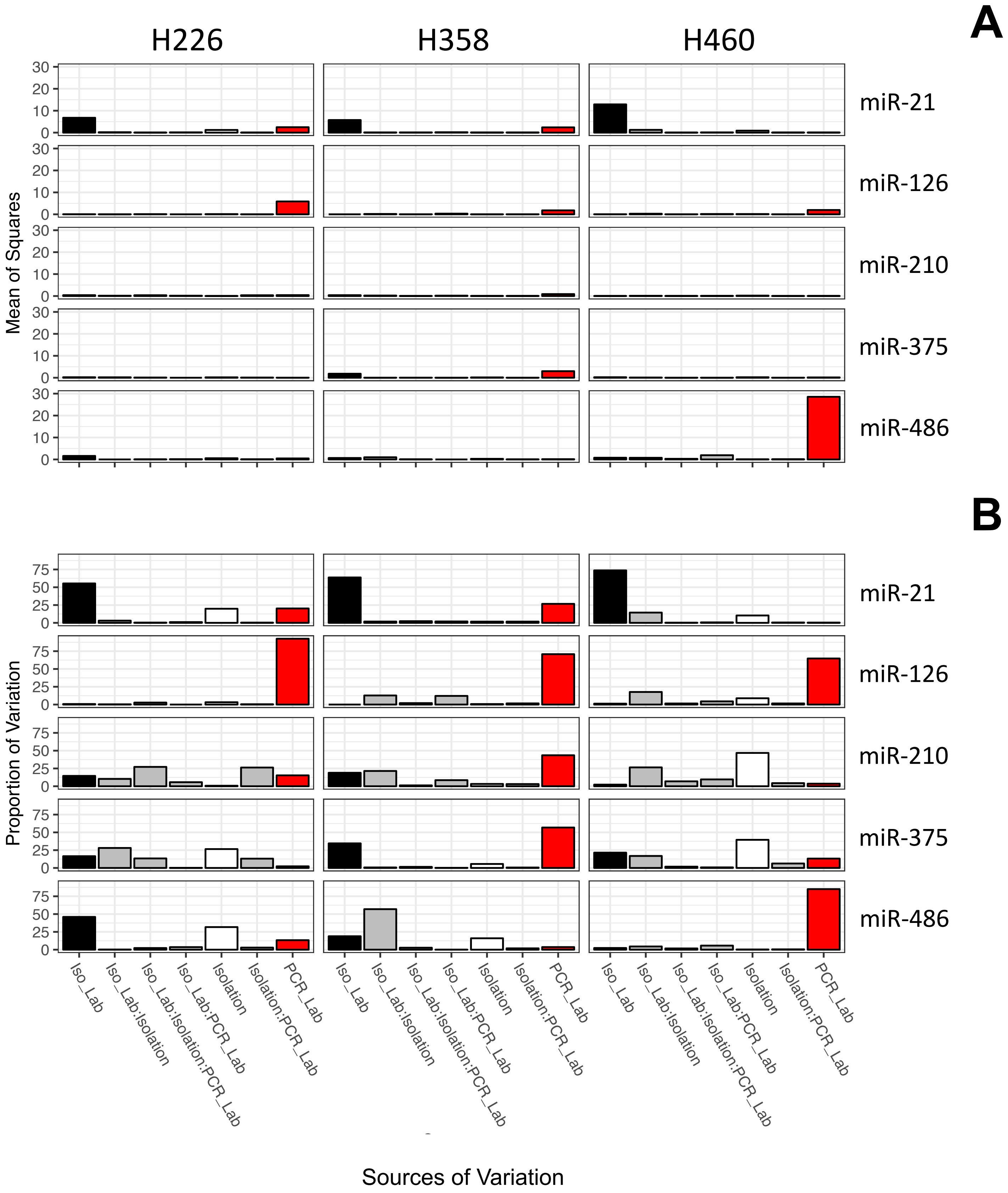

Supplemental Figure 1. Sources of variance in the miRNA profiling dataset. An analysis of variance in R[17] was used to identify the major sources of variability in the miRNA profiling for each cell line and analyte combination. A three-way ANOVA, testing all possibile interactions, was applied to determine the contribution to variance by isolation laboratory, isolation process, and PCR laboratory. The input data for the model was the Cq for each of the four measurement processes for three isolations. **Panel A** shows the mean of squares as a measure of the contribution of each factor or interaction to the variability in the experiment. **Panel B** shows the proportion of variation (sum of squares divided by total sum of squares) for each cell line miRNA combination.
